# Challenges in determining whether creativity and mental illness are associated

**DOI:** 10.3389/fpsyg.2015.00163

**Published:** 2015-02-20

**Authors:** Joscelyn E. Fisher

**Affiliations:** Department of Psychiatry, Uniformed Services University of the Health SciencesBethesda, MD, USA

**Keywords:** mental illness, methodological challenges, creativity, cognition, psychopathology

The existence of a relationship between “creativity” and unusual mental states has been speculated on for centuries, with a specific connection of “creativity” to “mental illness” since the 1830s (Becker, 2001). However, controversy remains about whether this relationship exists (e.g., Schlesinger, 2009). The main challenge in supporting this claim is that the statement itself is very general. In addition, there are a number of issues that contribute to unclarity within this literature.

One issue is the way in which “creativity” and “mental illness” are discussed. “Creativity” is a broad construct that has been defined and operationalized in various ways across the studies that have attempted to examine it. This variety is due to the fact that creativity is likely composed of various facets (Dietrich and Kanso, 2010), but has often been referred to as if it is a unitary construct (see Glazer, 2009, for discussion). Similarly, “mental illness” is a heterogeneous construct that not only encompasses multiple symptoms and diagnoses but reflects societal and cultural definitions and norms, resulting in changes to diagnostic criteria sets throughout the years. Researchers have tried to answer the question of whether a relationship between “creativity” and “mental illness” exists, but, as would be expected when tackling such a broad question, the approaches of each study have differed. In practice, both “creativity” and “mental illness” have been operationalized in every research study that has tackled this issue by recruiting a particular population and using a specific definition (whether articulated or not) of creativity in order to successfully examine the construct. But after the conclusions have been made, the titles and introductions of the next journal articles on the topic discuss the broad concepts of “creativity and mental illness” and/or cite references that studied one facet of creativity in a population to support an association with another facet of creativity in the same population without an explanation of why a similar finding would be expected. Thus, overlooking the details of what was actually studied in previous papers and drawing support from any study that refers to “creativity” even though it may represent a different facet of creativity makes it difficult to make clear-cut statements about a relationship between creativity and mental illness or even whether such a broad comparison is useful.

To foster examination of potential relationships between creativity and mental illness, it would be prudent to use a more systematic approach in which these constructs are made explicit in each study (Prentky, 2001). Given the diverse definitions and measures of creativity employed to date, a given study should focus on one of these definitions, describe why that definition is appropriate for study in a particular population, and use a measure that taps that particular facet of creativity. For example, Glazer (2009) proposed three possible models (1. different types of creativity each associated with a specific psychopathology, 2. creativity as a dimension, and 3. creativity as a unitary construct) for the creativity construct and how each would be associated with psychopathology. Using such a framework (or another that is similarly clearly defined) would expedite the process of answering the question about whether there is a relationship between mental illness and creativity.

Another issue that contributes to confusion in the field is the use of various “creativity measures” that measure different facets of creativity across studies. The results of individual studies are often generalized to an overall conclusion about “creativity” without discussion about how these facets may be related to each other. A goal in a given study would be to determine whether and how the creativity facet tested by the primary creativity measure is related to other frequently-used creativity measures, by including multiple measures of creativity. Using multiple creativity measures in one study would provide data for convergent and discriminant validity between the facets of creativity measured in that study.

A further step in defining the facet of creativity being studied would be to hypothesize whether additional cognitive mechanisms are relevant to the selected facet of creativity. Inclusion of cognitive measures (i.e., neuropsychological measures or behavioral tasks) that assess these mechanisms would allow determination of whether and how much creativity and cognitive measures overlap and allow integration and comparison of results to other literature that involves cognitive skills. For instance, Boden (2013) suggests that an understanding of associative pathways regarding semantic information and its relevance to context is important for creativity. Making semantic associations between words or concepts (likely associated with verbal creativity) has been related to executive function and positive schizotypy (Fisher et al., 2007, 2013), somewhat consistent with Eysenck's (1993) theory that psychoticism (P) is mediated by high divergent thinking (often referred to as an aspect of creativity) and low inhibition. Other facets of creativity are likely related to other cognitive processes (e.g., use of spatial relationships, problem solving, pattern recognition, cognitive inhibition). Incorporating cognitive measures from these fields could help shed light on what creativity is, how it works and whether there are multiple mechanisms that lead to the same facet of creativity.

In addition to both a more explicit definition and operationalization of creativity and investigating its associated cognitive correlates, a study would be clearer about why a certain type of mental illness is being investigated in relation to that type of creativity. Many studies have investigated a relationship between creativity and the schizophrenia spectrum (e.g., Weinstein and Graves, 2002; Fisher et al., 2004; Folley and Park, 2005), bipolar disorders (e.g., Soeiro-de-Souza et al., 2011), hypomania (e.g., Furnham et al., 2008), depression and anxiety (e.g., Silvia and Kimbrel, 2010), and autism characteristics (e.g., Rawlings and Locarnini, 2008; Claridge and McDonald, 2009). However, these diagnoses differ from each other in addition to having heterogeneous presentations of symptoms within each diagnosis. Thus, when considered as a whole, it is unclear why all of these disorders would be associated with creativity, especially if creativity is a unitary construct as it is often referred. Examining one facet of creativity in more than one mental illness or symptom type within one study could assist in determining specificity of that facet to a particular symptom type. It is more likely that one symptom or a number of symptoms in combination, either common or unique to multiple diagnoses, would be associated with a particular facet of creativity than an overall diagnosis or mental illness as a whole.

Furthermore, any facet of creativity is unlikely to be associated with clinical levels of symptoms. For instance, some of the most consistent findings about associations between performance on creativity measures and psychopathology-spectrum symptoms have been in samples of individuals with subclinical schizoprehnia-spectrum characteristics (undergraduates with high scores on schizotypy or first-degree relatives of those who have been diagnosed with a mental disorder) and not those diagnosed with schizophrenia (e.g., Jaracz et al., 2012). Thus, mental illness is likely an invalid term.

As a final note, a study that incorporates measures of creativity, cognition and symptoms may have to rely on statistical methods that do not assume linearity. Associations between cognitive skills and a facet of creativity, between cognitive skills and symptoms, and between a facet of creativity and symptom constellations are likely quite complex; thus, it is unlikely that an association between all three would be linear. To support this statement, there is evidence that schizotypy characteristics and executive function are curvilinearly associated with semantic processing in an inverted U-shape (Fisher et al., 2013). Similarly, Abraham (2014) suggested that top-down control and originality are associated in this manner. These studies are akin to Nelson and Rawlings (2010) suggesting that creativity increases with moderate schizotypy and decreases with increased more serious psychopathology and Stoneham and Coughtrey (2009) finding that high and low schizotypy groups are faster to solve a creative problem-solving task than those in an intermediate schizotypy group. Reliance on statistical methods designed to detect linear relationships may have contributed to the inconsistency of findings in the literature. The use of more sophisticated statistics that test the possibility of other types of associations between these constructs would allow better testing of more complex relationships.

In summary, it is difficult to answer whether there is a relationship between creativity and mental illness given the various methods and populations that have been studied in pursuit of this question. If a more detailed approach is used to engage this question more systematically, we may finally be able to put this age-old broad question to rest and instead ask more targeted ones.

## Disclaimer

The author's expressed opinions do not necessarily reflect those of the Uniformed Services University.

### Conflict of interest statement

The author declares that the research was conducted in the absence of any commercial or financial relationships that could be construed as a potential conflict of interest.
